# Screening for Light Personalities in Portugal: A Cross-Cultural Validation of the Light Triad Scale With an At-risk-of-delinquency Sample

**DOI:** 10.1177/0306624X241228234

**Published:** 2024-02-09

**Authors:** Pedro Pechorro, Makilim N. Baptista, Bruno Bonfá-Araujo, Cristina Nunes, Matthew DeLisi

**Affiliations:** 1 University of Coimbra, Coimbra, Portugal; 2 Pontifícia Universidade Católica de Campinas, Brazil; 3 University of Western Ontario, London, Canada; 4 Psychology Research Centre (CIP), Universidade do Algarve, Faro, Portugal; 5 Iowa State University, Ames, USA

**Keywords:** assessment, Light Triad Scale, measurement invariance, validation studies

## Abstract

The Light Triad of personality refers to three prosocial personality traits—Faith in Humanity, Humanism, and Kantianism—that promote the worth and dignity of other people, focus on ethical behavior and empathy, and confidence that other people are naturally good. The aim of the present study is to examine the psychometric properties of the Light Triad Scale (LTS)—Portuguese version. Our convenience sample consisted of 242 male and female participants (*M* = 30.19 years, *SD* = 12.78, range = 16–77) from Portugal. The proposed latent structure models of the LTS obtained adequate fits. Internal consistency/reliability, as measured by the alpha and omega coefficients, was adequate to good. Construct validity with other psychometric measures (i.e., empathy, dark traits of personality, propensity to morally disengage, and antisociality/criminality measures) and criterion-related validity (with justice involvement variables such as problems with the law, arrested by the police, sentenced to prison and alcohol/drug abuse variables) were demonstrated. Cross-gender measurement invariance was established, with females scoring higher than males. The findings support the use of the LTS as a valid and reliable measure.

## Introduction

Personality psychology contains a spectrum of theories and measures focused on comprehending the core aspects of human behavior and cognition. Recently, there has been a growing investment in the study of positive personality traits and their consequence on personal and societal well-being (Koydemir et al., 2021; Laborde et al., 2017; Musek & Grum, 2021), characteristics that from a criminological perspective might be human capital assets or protective factors that buffer an individual from antisocial behavior. One such construct that has attained notable engagement is the Light Triad (Kaufman et al., 2019). While the Light Triad has been mostly studied in English-speaking groups, its application and validity in different cultural contexts remain open to further investigation. Thus, in this article, we aim to fill this gap by adapting the Light Triad Scale to Portuguese and examining its psychometric properties and cross-cultural implications within the Portuguese-speaking community.

The Light Triad contains three factors: Faith in Humanity, Humanism, and Kantianism (Kaufman et al., 2019). Faith in Humanity entails an optimistic view of human nature, trust in others, and a belief in the prevailing good of Humanity. Humanism characterizes a belief in the intrinsic value and dignity of humans, emphasizing kindness, empathy, and helping others. Finally, Kantianism describes a person’s commitment to authenticity and moral principles. It was previously shown that positive traits are interrelated with other behaviors, such as empathy and prosociality (Kaufman et al., 2019; Stavraki et al., 2023; Ucar et al., 2023). Specifically, the Light Triad traits are associated with empathy, suggesting that people with high scores are characterized by compassion and willingness to help others and are likely to exhibit higher levels of empathy (Kaufman et al., 2019). This association can be explained by the fact that people who possess positive traits associated with the Light Triad are keen to consider and connect with the feelings and experiences of others, promoting an authentic respect for their well-being (Barros et al., 2022a).

On the other hand, prior investigations have shown that prosocial traits are negatively associated with aversive behaviors (Barros et al., 2022b; Lukić & Živanović, 2021). The Light Triad negatively correlated with dark traits such as Machiavellianism, narcissism, psychopathy, and sadism, collectively known as the Dark Tetrad. Particularly because the Kantianism trait represents honesty and treating others as ends onto themselves (i.e., lacking core features of the Dark Tetrad), therefore, this negative association can be attributed to the fact that the positive traits, especially those associated with the Light Triad, act as partially protective factors against the employment of socially undesirable behaviors and manipulative tendencies (Kaufman et al., 2019). However, some studies have shown that the overuse/underuse of positive characteristics also has a negative impact on human development (Barros et al., 2022b; Niemiec, 2019).

Moreover, the Light Triad, which encompasses ethical principles and positive regard for others, is probable to be inversely associated with violence and criminality. In this regard, it is a matter of considerable criminological importance. People with high scores in the Light Triad are expected to have lower dispositions toward violence because of the promotion of moral values and empathy (Ucar et al., 2023). Thus, these individuals may be less prone to commit criminal actions because of their internalized ethical standards, concern for others, and trust in the inherent goodness of people. This may happen because of the greater self-control shown by men and women with higher levels of prosocial behavior, exhibiting empathy-driven self-regulation to engage mostly in activities that will have desirable consequences. However, further investigation into how such behavior is associated is still needed.

To bring new information to this topic, the adaptation of measures that assess prosocial behavior is needed in different cultures, especially with the intent to provide a more diverse measurement and understanding of how they work in diverse groups. The Light Triad Scale, originally developed and validated in English, also with adequate results for other languages, such as Polish (Gerymski & Krok, 2019), Spanish (Stavraki et al., 2023), Brazilian-Portuguese (Barros et al., 2022a), and countries, such as the Philippines (Aruta, 2023), may exhibit variations in its factorial structure, item relevance, and psychometric properties when applied to a Portuguese-speaking population.

Gerymski and Krok (2019) observed gender differences in Polish participants, with women demonstrating higher scores across all Light Triad traits than men, yielding a medium effect size. Their three-factor model showed adequate results, incorporating covariance (i.e., CFI = .93, TLI = .91, RMSEA = .05). Notably, their findings suggested that an improved model fit could be achieved by excluding Kantianism (i.e., CFI = .97, TLI = .96, RMSEA = .04). This proposition stemmed from the possible misinterpretation of Kantianism items by Polish respondents, potentially suggesting nuanced variations between the US and Poland. Correspondingly, Stavraki et al. (2023) corroborated the three-model structure for Spanish participants with satisfactory fit indices (i.e., CFI = .94, TLI = .94, SRMR = .04, and RMSEA = .05). Acceptable adequacy was also observed for a two-factor model (i.e., Faith in Humanity and Humanism; CFI = .97, TLI = .97, SRMR = .04, and RMSEA = .05). Moreover, they established measurement invariance concerning gender and age. Aruta’s (2023) model yielded satisfactory outcomes for Filipino young adults, particularly in adherence to the three-factor configuration (i.e., CFI = .95, TLI = .93, SRMR = .04, and RMSEA = .05). Finally, Barros et al. (2022a) discerned no gender-based distinctions in Light Triad traits for Brazilian participants, and their model evaluation yielded adequate fit for the three-factor structure (i.e., CFI = .99 and RMSEA = .03). However, they noted incongruences in items associated with the Kantianism factor, showcasing heightened interrelations with the remaining two factors (i.e., Faith in Humanity and Humanism). By adapting the scale to Portuguese, we can ensure its applicability and contribute to a more comprehensive understanding of positive personality traits across cultures. It is worth mentioning the several existing differences between written and spoken Portuguese in Portugal and Brazil; thus, to ensure a reliable version of the measure, we opted to adapt the instrument from its original version.

We aim with this article to propose the cross-cultural exploration of the Light Triad Scale to the European Portuguese spoken in Portugal, a culturally distinct and linguistic context. The adaptation process involves not only linguistic translation but also the consideration of cultural nuances, values, and idiomatic expressions prevalent within the Portuguese-speaking community. We hypothesized that the LTS would show an adequate three-factor latent structure using confirmatory factor analysis and cross-gender measurement invariance, subscales would be significantly positively correlated, show adequate to good internal consistency/reliability, construct validity (with basic empathy, dark traits of personality, propensity to morally disengage, violence evaluation, low self-control, and antisociality/criminality tendencies measures), criterion-related validity (with trouble with the law, arrested by police, sentenced to prison, and alcohol/drug abuse variables), and known-groups validity (i.e., females scoring significantly higher on the LTS total score and Faith in Humanity, Humanism, and Kantianism subscales scores).

## Method

### Participants

The current convenience sample consisted of 242 participants (*M* = 30.19 years, *SD* = 12.78, range = 16–77), composed of females (*n* = 141, *M* = 28.93 years, *SD* = 12.61, range = 16–68) and males (*n* = 101, *M* = 31.96 years, *SD* = 12.87, range = 17–77 years). No significant differences were found between females and males regarding age (*F* = 3.34, *p* = .07, η_*p*_^2^ = .02) and ethnicity (χ^2^ = .91, *p* = .67, Φ = .12), but females reported having significantly higher education (χ^2^ = 46.12, *p* < .001, Φ = .44) and being significantly less frequently employed full-time (χ^2^ = 21.91, *p* < .001, Φ = .30). Males reported significantly higher levels of being in trouble with the law (χ^2^ = 73.93, *p* < .001, Φ = .55), of being arrested by the police (χ^2^ = 39.25, *p* < .001, Φ = .40), of being sentenced to prison (χ^2^ = 17.51, *p* < .001, Φ = .27), and of abusing alcohol and/or drugs (χ^2^ = 7.99, *p* = .007, Φ = .23).

### Measures

Light Triad Scale (LTS; Kaufman et al., 2019). This is a self-report measure of personality that includes three factors with 4 items each (that is, 12 items total), namely Faith in Humanity, Humanism, and Kantianism. All LTS items in the current study were formatted as 5-point Likert scales with anchors 1 (*Strongly disagree*) and 5 (*Strongly agree*). Factor scores are obtained by adding the respective items, and a total score can also be obtained by adding all the items. An increased prevalence of light traits is reflected in higher scores. The reliability for this study will be presented in the Results section.

Basic Empathy Scale—Adapted (BES-A; Jolliffe & Farrington, 2006). This is a self-report adapted short-form measure of basic empathy (7 items total) that includes two factors, namely, Affective empathy (3 items) and Cognitive empathy (4 items). All BES-A items in the current study were formatted as 5-point Likert scales with anchors 1 (*Strongly disagree*) and 5 (*Strongly agree*). Factor scores are obtained by adding the respective items, and a total score can also be obtained by adding all the items. An increased prevalence of empathy is reflected in higher scores. The BES-A Portuguese version was employed in the current study (Pechorro et al., 2017). Reliability for this study was BES-A total α = .83, Affective empathy α = .89, and Cognitive empathy α = .84.

Short Dark Tetrad (SD4; Paulhus et al., 2021). This is a self-report measure of personality that includes four factors with seven items each (i.e., 28 items total), namely Machiavellianism, Narcissism, Psychopathy, and Sadism. All SD4 items in the current study were formatted as 5-point Likert scales with anchors 1 (*Strongly disagree*) and 5 (*Strongly agree*). Factor scores are obtained by adding the respective items, and the use of a total score is not recommended by most authors. An increased prevalence of dark traits is reflected in higher scores. The SD4 Portuguese version was employed in the current study (Pechorro, Karandikar, et al., 2023). Reliability for this study was Machiavellianism α = .66, Narcissism α = .77, Psychopathy α = .79, and Sadism α = .81.

Dark Core of Personality (D16; Moshagen et al., 2020). This is a short self-report unidimensional version of the dark core of personality construct measure (16 items total). All D16 items in the current study were formatted as 5-point Likert scales with anchors 1 (*Strongly disagree*) and 5 (*Strongly agree*). A total score can be obtained by adding all the items. An elevated prevalence of dark personality is reflected in higher scores. The D16 Portuguese version was employed in the current study (Pechorro et al., in press). The reliability for this study was α = .79.

Low Self-Control Scale—Short Form (LSCS-SF; Grasmick et al., 1993). This is a self-report short version measure of low self-control (12 items in total), as defined in the general theory of crime (Gottfredson & Hirschi, 1990). All LSCS-SF items in the current study were formatted as 5-point Likert scales with anchors 1 (*Strongly disagree*) and 5 (*Strongly agree*). A total score can be obtained by adding all the items. An elevated prevalence of low self-control is reflected in higher scores. The LSCS-SF Portuguese version was employed in the current study (Pechorro, DeLisi, et al., 2023). The reliability for this study was α = .81.

Propensity to Morally Disengage Scale (PMDS; Moore et al., 2012). This is a self-report short unidimensional measure of the propensity to morally disengage (8 items total). All PMDS items in the current study were formatted as 5-point Likert scales with anchors 1 (*Strongly disagree*) and 5 (*Strongly agree*), while in the original study, items were formatted as 7-point Likert scales (Moore et al., 2012). A total score can be obtained by adding all the items. An elevated prevalence of propensity to morally disengage is reflected in higher scores. The PMDS Portuguese version was employed in the current study (Pechorro, Bonfá-Araujo, et al., 2024). The reliability for this study was α = .80.

Evaluation of Violence Questionnaire (EVQ; Nunes et al., 2021). This is a self-report unidimensional measure of violence evaluation (17 items total), that is, the extent to which one views violence (real or hypothetical) against other people as positive. All EVQ items in the current study were formatted as 4-point Likert scales with anchors 1 (*Very bad*) and 4 (*Very good*). A total score can be obtained by adding and averaging all the items. More positive violence evaluation is reflected in higher scores. The EVQ Portuguese version was employed in the current study (Nunes et al., in press). The reliability for this study was α = .94.

Antisociality Criminality Scale (ACS; Pechorro, Cordeiro, et al., 2023). This is a self-report unidimensional measure of antisociality and criminality tendencies (20 items total). All ACS items in the current study were formatted as 5-point Likert scales with anchors 1 (*Never*) and 5 (*Always or Almost always*). A total score can be obtained by adding all the items. An elevated prevalence of antisociality and criminality tendencies is reflected in higher scores. The original ACS Portuguese version was employed in the current study (Pechorro, Cordeiro, et al., 2023). The reliability for this study was α = .93.

A sociodemographic self-report questionnaire designed to measure the relevant sociodemographic variables (e.g., gender, ethnicity, education) was used to provide sociodemographic information and complement the psychometric instruments described above. This questionnaire also included some criminal questions regarding having been in trouble with the law as a defendant, having been arrested by the police, having been sentenced to prison, and abusing alcohol and/or drugs (dichotomously coded as 0 = *No* and 1 = *Yes*).

### Procedures

The cross-cultural translation and validation process of the LTS followed the recommended translation/back-translation procedure (American Educational Research Association [AERA] et al., 2014). The translation into the European Portuguese language was conducted, taking into consideration linguistic and conceptual issues. The subsequent back-translation was independently done by a native English speaker translator who was fluent in Portuguese. The original LTS and its back-translated version were then compared in terms of equivalence, and small adjustments were made. After this procedure, a pilot study was subsequently conducted to make sure the participants could easily understand the items. This concluded the translation/back-translation procedure of the Portuguese version of the LTS (available upon request).

The ethics committee granted authorization to conduct the present study. This study follows the ethical standards of the 1964 Declaration of Helsinki, including its later amendments. Our sample was a convenience one, partly collected online and partly collected using face-to-face interviews. The face-to-face interviews were mainly done with participants from impoverished and socially excluded zones of southern Portugal (e.g., Setúbal, Olhão), with limited economic resources and employment opportunities, characterized by having higher rates of criminality (Ferreira, 2011; Santana, 2002). A substantial part of the participants was contacted in social institutions managed by the Portuguese State (e.g., social welfare and reintegration institutions). After being informed about the current investigation, participants were asked to anonymously and voluntarily complete questionnaires. The usual mandatory informed consent was obtained from all of the participants. No monetary compensation or other forms of compensation were given for participating in the current study. Questionnaires with missing values were excluded from the study.

### Data Analyses

SPSS Statistics v28 software (IBM Corp., 2021) was used to conduct traditional psychometric analysis procedures. For example, descriptive statistics, Pearson and point-biserial correlations (low if <.20, high if >.50, moderate if in between), chi-square tests (with phi effect size—Φ; .10 is considered to be a small effect size, .30 a medium effect, and .50 a large effect), and ANOVAs (with power estimation and partial Eta squared effect size—η_*p*_^2^; .01 is considered a small effect size, .06 a medium effect, and .14 a large effect) (Ferguson, 2009; Maroco, 2021). Reliability was examined using the more traditional Cronbach’s alpha (α), item-total correlations (ITC; adequate if > .20), and mean item intercorrelations (MII; adequate if in the range .15–.50), but also McDonald’s omega (ω; marginal if > .60, adequate if > .70, good if > .80; excellent if > .90; Maroco, 2021; Simms & Watson, 2007). The use of omega is becoming increasingly recommended because it is a better estimator of reliability (Hayes & Coutts, 2020).

EQS v6.4 software (Bentler & Wu, 2018) was used to conduct Confirmatory Factor Analysis (CFA) procedures, employing the Maximum Likelihood Robust (MLR) estimator and correlation matrixes. That is, robust methodologies were used with polychoric correlations to perform the CFAs on the ordinal items because they provide more accurate estimates (Byrne, 2006). Mardia’s normalized estimate was higher than the cutoff value of 5, suggesting non-normality. We considered for an adequate model fit: Comparative Fit Index (CFI) and Incremental Fit Index (IFI) > .90, Robust Root Mean Square Error of Approximation (RMSEA 90% CI) < .08, and lowest Akaike Information Criterion (AIC) for a good model fit: CFI and IFI > .95, Robust RMSEA 90% CI < .06, and lowest AIC. We also considered the Satorra-Bentler scaling correction chi-square (SBχ^2^) and degrees of freedom (df). The sample size was mostly in accordance with the rule-of-thumb of at least a ratio of 10:1 (number of participants per number of items) when conducting CFAs (Kline, 2015) and was above the recommended minimum sample size of *N* = 100 necessary to obtain a potential statistical power level of .80 with, with a probability level of .05, and an anticipated potential effect size of .50 (Cohen, 1988; Soper, 2023; Westland, 2010; see also Sample Size Calculator for Structural Equation Models at https://www.danielsoper.com/statcalc/calculator.aspx?id=89). The recommended cutoff for the exclusion of items was a .40 or below standardized loading (Maroco, 2021).

We examined several different CFA models, namely: a one-factor model where all the items loaded on one factor; a three-factor model with intercorrelated factors where items loaded onto the three factors (Faith in Humanity, Humanism, and Kantianism); a second-order model with first-order uncorrelated factors where items loaded onto the three factors; and a bifactor model with first-order uncorrelated factors where items loaded onto the three factors and onto a general higher factor. No modification indices were used to improve the fits of the models. Measurement invariance was examined using ΔSBχ^2^(df), CFI, and RMSEA (90% C.I.), taking into consideration the recommended ΔCFI .01 cutoff and the ΔRMSEA (90% C.I.) .015 cutoff (Chen, 2007).

## Results

We began by doing a sociodemographic and justice-involvement characterization of the sample, as detailed in the Participants subsection. The effect sizes of the comparisons of the male and female participants regarding the sociodemographic and justice-involvement variables presented above can be mostly considered small to medium.

Table 1 shows the different goodness of fit indices obtained with regard to the CFA models examined. The three-factor intercorrelated model obtained the overall best fit, but the three-factor second-order model and the bifactor model also obtained adequate fits, legitimizing the use of the LTS total score.

**Table 1.** Fits for the Different Models of the LTS.

Models	SBχ^2^/*df*	IFI	CFI	RMSEA [90% CI]	AIC
Total sample					
1-factor	4.88	0.90	0.90	0.13 [0.11, 0.14]	156.71
3-factor intercorrelated	1.65	0.99	0.98	0.05 [0.03, 0.07]	−17.16
3-factor 2^nd^ order	1.98	0.93	0.93	0.06 [0.05, 0.08]	−0.49
3-factor bifactor	2.05	0.94	0.94	0.07 [0.05, 0.09]	2.06
Male sample					
1-factor	2.74	0.69	0.68	0.13 [0.11, 0.16]	40.13
3-factor intercorrelated	1.04	0.99	0.99	0.02 [0.00, 0.06]	−49.02
3-factor 2^nd^ order	1.34	0.95	0.94	0.06 [0.00, 0.09]	−32.70
3-factor bifactor	1.39	0.95	0.95	0.06 [0.00, 0.10]	−25.48
Female sample					
1-factor	3.16	0.72	0.71	0.13 [0.10, 0.15]	63.23
3-factor intercorrelated	1.16	0.99	0.99	0.03 [0.00, 0.07]	−42.68
3-factor 2^nd^ order	1.54	0.94	0.93	0.06 [0.03, 0.09]	−23.19
3-factor bifactor	1.56	0.95	0.95	0.06 [0.03, 0.09]	−19.49

*Note*. LTS = Light Triad Scale.

Table 2 displays the CFA item loadings for the three-factor intercorrelated model of the LTS. All items loaded above the .40 recommended cutoff.

**Table 2.** Standardized Loadings for the LTS.

Items	Loadings
Faith in humanity	
1. I tend to see the best in people.	0.78
2. I tend to trust that other people will deal fairly with me.	0.82
3. I think people are mostly good.	0.73
4. I’m quick to forgive people who have hurt me.	0.42
Humanism	
5. I tend to admire others.	0.58
6. I tend to applaud the successes of other people.	0.81
7. I tend to treat others as valuable.	0.91
8. I enjoy listening to people from all walks of life.	0.70
Kantianism	
9. I prefer honesty over charm.	0.79
10. I don’t feel comfortable overtly manipulating people to do something I want.	0.86
11. I would like to be authentic even if it may damage my reputation.	0.58
12. When I talk to people, I am rarely thinking about what I want from them.	0.73

*Note*. LTS = Light Triad Scale.

Table 3 shows the cross-gender invariance analysis of the LTS, which revealed the presence of both weak and strong measurement invariance. That is, the ΔSBχ^2^(df) were non-significant, and the ΔCFI and the ΔRMSEA were below the recommended cutoffs (Chen, 2007).

**Table 3.** Cross-Gender Invariance of the LTS.

Model	SBχ^2^ (*df*)	ΔSBχ^2^ (*df*)	CFI	RMSEA [90% CI]
Configural	113.24 (102)	—	0.99	0.03 [0.00, 0.06]
Weak/metric	126.41 (111)	12.76 (9)^ns^	0.99	0.03 [0.00, 0.06]
Strong/scalar	133.58 (117)	19.62 (15)^ns^	0.99	0.03 [0.00, 0.06]

*Note*. LTS = Light Triad Scale; ns = non-significant.

Table 4 shows the intercorrelations of LTS subscales across the total sample and the male and female samples. As expected, the intercorrelations were positive, moderate to strong, and statistically significant (Ferguson, 2009), showing consistencies between the total sample and the male and female samples. The strongest association between the factors was between Humanism and Kantianism, and the three factors tended to present similar associations to the LTS total.

**Table 4.** Pearson Intercorrelations of the LTS Total and Subscales.

	LTS total	Faith in humanity	Humanism	Kantianism
Total sample				
LTS total	1			
Faith in humanity	0.78***	1		
Humanism	0.79***	0.39***	1	
Kantianism	0.78***	0.33***	0.54***	1
Male/Female samples				
LTS total	1			
Faith in humanity	0.74***/0.81***	1		
Humanism	0.81***/0.75***	0.39***/0.37***	1	
Kantianism	0.75***/0.77***	0.25***/0.37***	0.52***/0.51***	1

*Note*. LTS = Light Triad Scale.

*** *p* < .001.

Table 5 shows the internal consistency/reliability values across the total sample and the male and female samples. These values were adequate to good, as the LTS factors always presented alpha and omega values above .70, and the LTS total always presented alpha and omega values above .80 (Hayes & Coutts, 2020).

**Table 5.** Internal Consistency/Reliability of the LTS.

	LTS total	Faith in humanity	Humanism	Kantianism
Total sample				
α	0.84	0.75	0.80	0.76
ω	0.85	0.76	0.81	0.77
ITC	0.30, 0.65	0.37, 0.60	0.52, 0.72	0.47, 0.61
MII	0.31	0.42	0.50	0.45
Male/female samples				
α	0.84/0.83	0.71/0.76	0.83/0.75	0.76/ 0.74
ω	0.85/0.84	0.73/0.77	0.84/0.75	0.77/ 0.75
ITC	0.23, 0.70/0.35, 0.60	0.32, 0.60/0.40, 0.64	0.52, 0.79/0.47, 0.63	0.45, 0.63/0.40, 0.67
MII	0.30/0.29	0.38/0.44	0.55/0.42	0.44/0.41

*Note*. LTS = Light Triad Scale.

Table 6 shows the correlations of LTS with other psychometric measures and criteria used to establish external validity. Construct validity patterns were examined with basic empathy, dark traits of personality, the propensity to morally disengage, violence evaluation, low self-control, and antisociality/criminality tendencies, and criterion-related validity patterns were examined with the trouble with the law, arrested by police, sentenced to prison, and alcohol/drug abuse variables. The strongest construct validity associations were with the dark core of personality measure, and the only non-significant association was between the Faith in Humanity factor and the propensity to morally disengage measure. In terms of criterion-related validity, the only LTS factor presenting significant associations was the Kantianism factor.

**Table 6.** Construct-Related and Criterion-Related Validities of the LTS.

	LTS total	Faith in humanity	Humanism	Kantianism
BES-A total	0.49***	0.32***	0.45***	0.41***
BES-A Affective	0.40***	0.31***	0.35***	0.28***
BES-A Cognitive	0.41***	0.20**	0.40***	0.40***
SD4 Machiavellianism	−0.32***	−0.21**	−0.24***	−0.33***
SD4 Narcissism	−0.26***	−0.22**	−0.17**	−0.22***
SD4 Psychopathy	−0.32***	−0.15*	−0.25***	−0.36***
SD4 Sadism	−0.38***	−0.28***	−0.34***	−0.28***
D16	−0.57***	−0.31***	−0.50***	−0.59***
PMDS	−0.27***	−0.03	−0.30***	−0.37***
EVQ	−0.26***	−0.14*	−0.26***	−0.25***
LSCS-SF	−0.38***	−0.22**	−0.33***	−0.38***
ACS	−0.28***	−0.16*	−0.20**	−0.32***
Trouble with the law	−0.14*	0.01	−0.09	−0.21*
Arrested by police	−0.09	0.00	−0.06	−0.19*
Sentenced to prison	−0.04	0.06	−0.05	−0.14*
Alcohol/drug abuse	−0.13*	−0.10	−0.08	−0.15*

*Note*. LTS = Light Triad Scale; BES-A = Basic Empathy Scale—Adapted; SD4 = Short Dark Tetrad; D16 = Dark Core of Personality Very Short; PMDS = Propensity to Morally Disengage; EVQ = Evaluation of Violence Questionnaire; LSCS-SF = Low Self-Control Scale—Short; ACS = Antisociality Criminality Scale.

* *p* < .05. **p < .01. ***p < .001.

Lastly, Table 7 displays the total score and subscale means of the LTS and the respective gender comparisons. Differences emerged regarding the LTS total score and the Faith in Humanity, Humanism, and Kantianism subscales scores, with females obtaining significantly higher scores. The η_*p*_^2^ effect sizes of these comparisons of the male and female participants can be mostly considered small to medium (Olejnik & Algina, 2003).

**Table 7.** Known-Groups Gender Comparisons of the LTS.

	Male *M* (*SD*)	Female *M* (*SD*)	*F*, *p-*Value	η_*p*_^2^, power
LTS total	44.44 (6.46)	47.60 (6.04)	15.11, <.001	0.06, 0.97
Faith in humanity	12.92 (3.06)	13.78 (3.24)	4.31, 0.03	0.02, 0.54
Humanism	15.55 (2.64)	16.78 (2.12)	16.08, <.001	0.06, 0.98
Kantianism	15.93 (2.70)	17.10 (2.42)	12.44, <.001	0.05, 0.94

*Note*. LTS = Light Triad Scale.

## Discussion

The aim of this study was to validate the Portuguese version of the Light Triad Scale. To this end, several psychometric analyses of internal factor structure, measurement invariance, reliability, and evidence-based on the relationship with other variables (i.e., construct-related and criterion-related) were carried out.

The factor structure of the LTS was examined, revealing that the three-factor intercorrelated model, composed of the Faith in Humanity, Humanism, and Kantianism factors, obtained the overall best fit, which is consistent with most previous findings (Aruta, 2023; Gerymski & Krok, 2019; Kaufman et al., 2019; Lukić & Živanović, 2021). However, the three-factor second-order model and the bifactor model also obtained adequate fits, thus providing some legitimization of the use of a total score (Bornovalova et al., 2020; Brunner et al., 2012).

Analysis of the measurement invariance of the LTS across genders revealed the presence of weak and strong invariance, in line with previous studies (e.g., Stavraki et al., 2023). Establishing measurement invariance is crucial to legitimizing comparisons between different groups (Putnick & Bornstein, 2016). The correlation matrix of the LTS and its factors revealed the expected positive, moderate to strong significant associations, also mostly consistent with previous studies (e.g., Aruta, 2023; Gerymski & Krok, 2019; Kaufman et al., 2019; Stavraki et al., 2023).

When considering reliability, alpha and omega coefficients across the samples revealed adequate to good values. The LTS total was always above .80, and the Faith in Humanity, Humanism, and Kantianism factors were almost always above .70. The corrected item-total correlations and mean inter-item correlations results can be considered very good, as they were above the .30 cutoff value and within the .15 to .50 range, respectively. Our reliability results were similar to those found by Kaufman et al. (2019) and somewhat higher than the ones found by Gerymski and Krok (2019), Lukić and Živanović (2021), Stavraki et al. (2023), Ucar et al. (2023), and Aruta (2023). In scales with a reduced number of items in the factors, it is essential to have items balancing higher factor loadings and adequate reliabilities.

The construct validity with basic affective and cognitive empathy revealed the expected positive, moderate to strong statistically significant correlations, in line with previous research (e.g., Kaufman et al., 2019). Interestingly, Lukić and Živanović (2021) suggest that the Light Triad is not merely characterized by the presence of empathy for other people but by a clear repulsion against dark, sadistic tendencies. Criminologically, this suggests that Light Triad features are an asset or protective factor against antisocial behavior. Associations with dark traits of personality, the propensity to morally disengage, violence evaluation, low self-control, and antisociality/criminality tendencies measures demonstrated mostly negative, moderate to strong, and statistically significant associations. These associations were mostly in line with previous research (e.g., Kaufman et al., 2019; Lukić & Živanović, 2021; Ucar et al., 2023). Associations were particularly strong (mostly above −.50) with the D16 dark core of personality measure. Considering that Light Triad traits can be seen as contrasting dark traits of personality, this was to be expected (Kaufman et al., 2019).

The criterion-related validity of the LTS and its factors was examined with justice-involvement and alcohol/drug abuse variables. Mostly negative, low-significant associations were found, suggesting light personality traits do not continue through to the justice system. The notable exception to the trend pertained to Kantianism, which had negative low to moderate significant correlations with the trouble with the law, arrested by police, sentenced to prison, and alcohol/drug abuse variables. Research investigating the associations between light triad traits and antisocial/criminal variables is still very scarce or even non-existent, but considering the positive relations between light traits and prosocial outcomes (see e.g., Barros et al., 2022a; Gerymski & Krok, 2019; Kaufman et al., 2019; March & Marrington, 2023; Ruel et al., 2023), one could expect these null or negative correlations that were obtained. Light Triad features thus provide another avenue in criterion-validity analyses to assess the broader effects of personality and behavioral features.

Lastly, known-groups validity was demonstrated, with females scoring significantly higher than males on the LTS total and its subscales. This is in line with previous studies examining the LTS (e.g., Gerymski & Krok, 2019). Our findings corroborate the notion that these gender differences in light traits are indeed factual, not the result of measurement problems/artifacts. That women have higher LTS scores is compatible with sex differences favoring women in terms of antisocial personality and psychopathic features.

Regarding the use of an LTS total score, one could argue that it should be based on a conjunction of findings that include both factor analysis and construct validity. Our findings suggest that the factor analytic results provide some legitimization for it and that the three LTS subscales tend to show somewhat similar associations in terms of construct validity with the selected constructs, which is an argument in favor of the more parsimonious focus on the total score. However, the correlations among subscales are mostly moderate, and regarding the variables used to examine criterion-related validity (i.e., justice involvement and alcohol/drug abuse), only the Kantianism subscale shows significant and consistent associations. Thus, a strict focus on total score would hinder an appreciation of these nuances. These mixed findings lead us to conclude that an LTS total score should be used with caution, and one must also keep in mind that most previous studies did not provide evidence to support it.

### Limitations

We must consider some limitations of the current investigation. An initial important limitation we must mention is that we only used self-report data, which can be affected by common method bias and social desirability. Access to additional data sources and using a multi-method and multi-informant approach would have been a better option. Another limitation was that our sample was a mere convenience sample that included participants from impoverished and socially excluded zones of Portugal that are not strictly representative of the general Portuguese population, that is, that may hinder the generalization of results. Future studies should consider using additional psychometric procedures (e.g., item response theory), longitudinal research methodology, and more specific and representative types of samples (e.g., forensic, youth). Finally, it would also be interesting, in future studies, to investigate measurement invariance between different cultures in order to assess whether linguistic understanding and more specific cultural characteristics may interfere with responses to the LTS.

## Conclusion

In sum, our study demonstrated that the LTS is a valid and reliable measure of the light traits of personality construct. LTS total score and three-factor scores—Faith in Humanity, Humanism, and Kantianism—can be used for research purposes. The use of LTS is particularly recommended for applied settings, where brief measures are particularly useful. Although our findings are premature in terms of generating specific benevolent and beneﬁcent prosocial orientations policy suggestions, programs that bolster interpersonal skills and a more humane, empathic manner of engaging in interpersonal exchange can result in behavioral improvements.
